# The Role of Self-Compassion and Body Perception in Predicting Psychological Safety

**DOI:** 10.3390/jcm15135177

**Published:** 2026-07-02

**Authors:** Andrea Poli, Mario Miccoli

**Affiliations:** Department of Human and Social Sciences, Mercatorum University, 00186 Rome, Italy

**Keywords:** psychological safety, body perception, self-compassion, polyvagal theory, neuroception, psychological trauma, PTSD

## Abstract

**Background/Objectives**: Psychological safety is increasingly recognized as a clinically relevant construct linked to emotional regulation, interpersonal functioning, and trauma-related processes. Emerging evidence suggests that psychological safety may depend not only on embodied experiences of bodily regulation but also on how individuals relate to themselves under conditions of distress. The present study investigated the role of self-compassion and body perception on psychological safety. **Methods**: A total of 332 community participants were administered the Neuroception of Psychological Safety Scale (NPSS), the Body Perception Questionnaire-22 (BPQ-22), the Self-Compassion Scale-Short Form (SCS-SF), and the Depression Anxiety Stress Scale-21 (DASS-21). Spearman’s correlations, hierarchical multiple regression analyses, and mediation analyses were conducted using SPSS to examine the relationships among the study variables. **Results**: Psychological safety showed a moderate positive association with self-compassion and a weak negative association with body perception. Body perception was also negatively associated with self-compassion. In hierarchical regression models, self-compassion emerged as the strongest predictor of psychological safety, whereas the predictive effect of body perception became non-significant after self-compassion entered the model. Mediation analyses further demonstrated that self-compassion fully mediated the association between body perception and psychological safety. **Conclusions**: The findings suggest that the relationship between bodily experience and psychological safety may primarily depend on individuals’ capacity to respond to internal experiences with compassion directed towards oneself. Self-compassion may therefore represent a clinically relevant target for interventions aimed at enhancing psychological safety.

## 1. Introduction

Psychological safety is increasingly emerging as a clinically meaningful construct because it reflects a person’s capacity to experience the self, the body, and the interpersonal environment as sufficiently safe to sustain openness, affect regulation, and adaptive engagement [1,2,3,4,5]. Although the construct has often been examined in organizational and relational research, its relevance to clinical psychology and psychosomatic functioning is likely broader [6,7]. In clinical contexts, psychological safety may shape whether individuals are able to regulate distress, seek support, tolerate vulnerability, and engage in treatment without excessive defensiveness or threat anticipation. This fact may be especially relevant in trauma-informed care, where the experience of safety is not peripheral, but foundational to therapeutic access and psychological recovery [8].

Recent psychophysiological approaches suggest that psychological safety should not be conceptualized solely as a subjective appraisal of the environment, but also as an embodied state supported by neurophysiological processes that signal protection, soothing, and connectedness [9,10]. In the polyvagal framework [11,12], safety depends on neuroception, namely the largely implicit evaluation of cues of safety, danger, or life threat [11,13]. When safety is detected, physiological, emotional, and cognitive processes that support social engagement become more accessible; when danger predominates, defensive mobilization or shutdown states are more likely to prevail [14,15]. In this sense, psychological safety can be viewed as a functional psychobiological condition that enables relational trust, emotional flexibility, and behavioral regulation [3,16,17,18,19,20]. The polyvagal theory framework is directly reflected in the Neuroception of Psychological Safety Scale (NPSS), which was originally developed to assess psychological safety as a multidimensional construct integrating bodily sensations, compassion, and social engagement [21]. The NPSS is especially relevant because it moves beyond narrow interpersonal definitions and operationalizes psychological safety as a core construct within a soothing-contentment system, linking physiological regulation with affiliative and affective functioning [6,7,21]. In the Italian validation study, the NPSS also showed positive associations with self-compassion and negative associations with body perception-related distress, suggesting that psychological safety may lie at the intersection of embodied regulation and compassionate self-relating [22].

Among the psychological variables that may be most relevant to psychological safety, self-compassion [23] may be of particular interest. Self-compassion refers to a positive and non-judgmental attitude toward oneself in moments of failure, inadequacy, or suffering, and is classically articulated into the dimensions of self-kindness versus self-judgment, common humanity versus isolation, and mindfulness versus over-identification [23,24]. Rather than reflecting simple self-soothing, self-compassion captures a regulatory stance through which distress can be acknowledged without being amplified by shame, harsh self-criticism, or emotional over-identification. Accordingly, self-compassion may represent a key internal condition through which safety is not only perceived, but also maintained [25]. From a clinical perspective, the possibility of maintaining psychological safety is particularly relevant. If psychological safety reflects the ability to remain open and regulated in the face of challenge, then self-compassion may represent one of the most important mechanisms through which individuals metabolize distress without converting it into a threat. Self-compassion is relevant in contexts where the assessment of a positive attitude toward oneself is of interest, and in patients who may benefit from developing a compassionate self [22,26,27,28]. Taken together, these findings support the idea that self-compassion is not merely correlated with well-being, but may play a mechanistic role in shaping the experience of safety itself.

At the same time, any clinically meaningful model of psychological safety may remain inaccurate if the body is not taken into consideration as part of the research examination. A growing literature has shown that constitutional differences in the control and perception of bodily states may contribute to vulnerability to psychological symptoms [29,30]. Interoceptive and autonomic processes have been implicated across a wide range of conditions, including anxiety, depression, post-traumatic stress, eating disorders, psychosomatic syndromes, and other dysregulation-related presentations [31,32]. From this perspective, the body is not simply the background of psychological life; it is one of the main channels through which safety, threat, and dysregulation are experienced [33,34]. Body perception is therefore a particularly relevant candidate in the study of psychological safety. The Body Perception Questionnaire-22 (BPQ-22) was validated in Italian [35] from the original version [36] within a neurophysiologically informed framework to assess body awareness and autonomic reactivity, including supradiaphragmatic and subdiaphragmatic features. Importantly, the BPQ-22 does not merely index symptom-specific bodily complaints; it captures broader individual differences in the subjective experience of bodily signals and autonomic activation. As such, it offers a valuable way to investigate how embodied sensitivity and physiological reactivity may shape psychological functioning across both clinical and non-clinical settings [37].

This body-based perspective is highly relevant to the study of psychological safety. The three BPQ-22 dimensions showed weak but significant negative correlations with psychological safety measured by NPSS, suggesting that greater unpleasant bodily awareness and autonomic reactivity are associated with lower perceived safety [22]. The authors further note that BPQ-22 items are largely oriented toward unpleasant bodily sensations, and that low levels of such bodily discomfort may be necessary for psychological safety to emerge [22]. This finding is clinically coherent: when bodily signals are experienced as dysregulated, alarming, or difficult to interpret, the organism may be less able to access a state of calm connection and more likely to orient toward vigilance and defense [38,39]. The relationship between body perception and psychological safety may extend beyond a direct association. Distressing or dysregulated bodily experiences may be accompanied by self-judgment, catastrophic appraisal, and emotional over-identification, whereas self-compassion may promote balanced awareness and non-defensive acceptance. Self-compassion may therefore represent a theoretically plausible statistical mediator between bodily experience and psychological safety [40,41,42,43], consistent with its association with positive self-relating and emotionally balanced processing of emotional distress [44,45].

Accordingly, the present study was designed to examine the role of body perception and self-compassion in predicting psychological safety. Specifically, we investigated whether body perception, as measured by the BPQ-22 [35], is associated with neuroception-based psychological safety, as measured by the NPSS [22], and whether this association is mediated by self-compassion, assessed through the Self-Compassion Scale-Short Form [46]. This rationale is grounded in the convergence of three lines of evidence: first, psychological safety appears to be an embodied construct rooted in neurophysiological regulation [9,10]; second, body perception is associated with the quality of internal bodily experience and autonomic reactivity [47,48]; third, self-compassion appears theoretically and psychometrically linked to the development of safety-related states [25,44,49]. Within this framework, we expected body perception to be associated with psychological safety, but also hypothesized that this association would be fully accounted for by self-compassion. Hence, the present research aims to specifically achieve the following objectives: (a) examine the bivariate associations of NPSS with SCS-SF and BPQ-22; (b) examine how NPSS, SCS-SF, and BPQ-22 are differentially predicted by all the other variables of the study, carrying out hierarchical multiple regression; (c) examine a mediation model [50,51] where BPQ-22 is considered the independent variable, NPSS is considered the dependent variable, and SCS-SF is considered the mediator; (d) examine whether SCS-SF may be able to act as a partial, or total, mediator of the effects between BPQ-22 and NPSS.

## 2. Materials and Methods

### 2.1. Participants

Three hundred and thirty-two community individuals who responded to SurveyMonkey (SurveyMonkey Inc., San Mateo, CA, USA) email invitations were recruited as a non-clinical convenience sample for the present study in order to complete a set of psychological questionnaires. The response rate was approximately 18%. Participants under 18 years of age were excluded. Eligibility criteria included age ≥ 18 years, sufficient knowledge of Italian, and informed consent. Incomplete questionnaires were excluded; missing data were not imputed, and complete-case analyses were conducted on the final sample of 332 participants. The health status of patients was not collected. Although the present study relied on a non-clinical convenience sample, it has been shown that research carried out in non-clinical populations is able to provide valuable insights, particularly when examining dimensional psychological constructs [52]. Most participants were female (80.1%), and the mean age of the sample was 43.79 years (SD = 11.42), with ages ranging from 19 to 76 years. In terms of educational attainment, the majority of respondents (80.42%) had completed a Ph.D. or a post-graduate specialization, while 15.36% held a university degree (bachelor’s or master’s level), and 4.22% had completed secondary education. With regard to occupational status, most participants (79.22%) were employed, whereas 3.31% were undergraduate students, and 17.47% were unemployed, homemakers, or retired. Concerning relationship status, 58.73% were married or living with a partner, 33.73% were single, 6.93% were divorced, and 0.61% were widowed.

### 2.2. Measures

*Neuroception of Psychological Safety Scale (NPSS; [21])*. The NPSS is a self-report tool developed by Morton et al. [21] in order to assess neuroception of psychological safety. It comprises 29 items rated on a 5-point Likert scale ranging from 1 (“strongly disagree”) to 5 (“strongly agree”). Respondents are asked to indicate the extent to which they agree with each statement. The scale has been shown to include three dimensions: Social Engagement (SE), reflecting experiences of interpersonal safety (e.g., “There was someone who made me feel safe”); Compassion (COM), capturing compassionate feelings toward others (e.g., “I felt compassion for others”); and Bodily Sensations (BS), referring to perceived physiological steadiness and regulation (e.g., “My breathing was steady”). In our investigation, NPSS showed an α = 0.92.

*Body Perception Questionnaire-22 (BPQ-22; [35]).* Originally introduced by Porges [53] and subsequently refined by Cabrera et al. [36] and by Poli et al. [35], the BPQ is a self-report measure designed to evaluate body awareness and autonomic reactivity. In the present study, the 22-item Italian version was administered [35]. Participants rate how frequently they notice bodily sensations and autonomic responses using a 3-point scale ranging from 1 (“never”) to 3 (“often”). The questionnaire includes three subscales: Body Awareness (BOA), which assesses awareness of physical sensations (e.g., “Watering or tearing of my eyes”); Supradiaphragmatic Reactivity (SUP), which captures autonomic symptoms above the diaphragm (e.g., “When I am eating, I have difficulty talking”); and Subdiaphragmatic Reactivity/Body Awareness (BOA/SUB), which refers to bodily and autonomic experiences below the diaphragm (e.g., “After eating I have digestive problems”). In our investigation, BPQ-22 showed an α = 0.86.

*Self-Compassion Scale-Short Form (SCS-SF; [54]).* Self-compassion was assessed using the SCS-SF [54], a 12-item self-report measure examining the tendency to respond to personal suffering with warmth, care, and a sense of connectedness. The instrument includes six components: self-kindness, self-judgment, common humanity, isolation, mindfulness, and overidentification. Participants are asked to indicate how they typically relate to themselves in difficult situations using a 5-point scale ranging from 1 (“almost never”) to 5 (“almost always”). Examples of items include statements such as “When I fail at something important to me, I become consumed by feelings of inadequacy” and “When something upsets me, I try to keep my emotions in balance.” In this study, the Italian translation by Poli et al. [46] was used. The scale showed good internal consistency in the present study (α = 0.89).

*Depression Anxiety Stress Scale-21 (DASS-21; [55,56,57]).* The DASS-21 is a 21-item self-report questionnaire measuring the core symptoms of depression, anxiety, and stress. The depression subscale assesses features such as reduced motivation, low self-worth, and dysphoria (e.g., “I couldn’t seem to experience any positive feeling at all”); the anxiety subscale evaluates both subjective and somatic manifestations of anxiety, as well as acute fear responses (e.g., “I felt scared without any good reason”); and the stress subscale captures irritability, tension, agitation, and difficulty relaxing (e.g., “I tended to over-react to situations”). Respondents rate the extent to which each statement applied to them over the previous week on a 4-point scale ranging from 1 (“Did not apply to me at all”) to 4 (“Applied to me very much or most of the time”). In the present study, the Italian version was validated by Bottesi et al. [58] was used. In our study, Cronbach’s α of the three DASS-21 subscales was as follows: α = 0.92 for the depression subscale, α = 0.86 for the anxiety subscale, and α = 0.87 for the stress subscale. For the DASS-21 total score, α = 0.95.

### 2.3. Procedure

After providing informed consent, participants received a brief explanation of the study aims and were then invited to complete a battery of self-report instruments, including the measures described above. To minimize potential order effects, the questionnaires were administered in a counterbalanced sequence. Completion of the full assessment battery required approximately 15 to 25 min. A research assistant remained available throughout the procedure to ensure that all questionnaires were completed and to clarify any items when necessary. Participation was voluntary, and no incentives were provided.

### 2.4. Statistical Analysis

All statistical analyses were performed with SPSS^®^ 27 (IBM Corp., Armonk, NY, USA). The Shapiro–Wilk test was performed to verify the non-normality of the distributions. Spearman’s zero-order correlations were first calculated to examine associations among the NPSS, BPQ-22, SCS-SF, and DASS-21 subscales and their total scores, and to evaluate the hypothesized relationships between study variables. Correlation coefficients were interpreted according to Cohen’s criteria [59], whereby values ranging from 0.70 to 0.89 were considered to be very strong, values ranging from 0.50 to 0.69 were considered strong, values between 0.30 and 0.49 were considered moderate, and values between 0.10 and 0.29 were regarded as weak. Bias-corrected and accelerated bootstrap (BCa) 95% confidence intervals based on 5000 resamples were calculated for Spearman’s correlation coefficients. To further assess the stability of these associations and to determine whether body perception, neuroception of psychological safety, and self-compassion scores may be explained by all of the other variables beyond the effects of depression, anxiety, and stress symptoms, three hierarchical multiple regression models were estimated. The three variables (body perception, neuroception of psychological safety, and self-compassion scores) were examined separately as dependent variables in each of the three hierarchical models. In each regression, the three subscale scores of the DASS-21 were entered in the first step as control variables. Finally, mediation analyses were conducted using the PROCESS macro [50,51] to test whether self-compassion scores mediated the association between body perception scores and neuroception of psychological safety scores. (BCa) 95% confidence intervals were calculated for regression and mediation analyses. In addition, in order to examine whether the mediation model was structurally equivalent across gender and educational groups, multi-group mediation analyses were conducted. An unconstrained model was compared with a constrained model in which all structural regression paths were set equal across groups. Following the recommendations by Ryu [60] and Ryu and Cheong [61], multi-group equivalence was evaluated primarily using a likelihood-ratio test of the equality constraints, adopting a significance level of α = 0.05. A non-significant test (*p* ≥ 0.05) was interpreted as indicating that the equality constraints did not significantly worsen model fit; therefore, supporting the structural equivalence of the mediation paths across groups. The absolute fit of the constrained model was additionally evaluated using comparative fit index (CFI), Tucker–Lewis index (TLI), root mean square error of approximation (RMSEA), and standardized root mean square residual (SRMR), with CFI and TLI values ≥ 0.95, RMSEA ≤ 0.05, and SRMR ≤ 0.08 considered indicative of a good model fit [62]. These cut-offs were interpreted as general guidelines rather than strict decision rules [63].

## 3. Results

### 3.1. Descriptive Statistics

Table 1 presents the descriptive indices for each measure, including mean, standard deviation, median, interquartile range, observed range, and Cronbach’s alpha coefficients. Overall, the average scores obtained in the present sample were consistent with normative values previously reported in Italian clinical samples (e.g., [22,35,46]).

### 3.2. Zero-Order Correlations

Table 1 also reports the zero-order Spearman correlations among the NPSS, the BPQ-22, the SCS-SF, and the DASS-21 total and subscale scores. In line with expectations, NPSS scores showed moderate positive associations with SCS-SF scores (ρ = 0.49; [0.397–0.573]; *p* < 0.01), and weak negative associations with BPQ-22 scores (ρ = −0.20; [−0.301–−0.085]; *p* < 0.01). SCS-SF scores showed weak negative associations with BPQ-22 scores, as well (ρ = −0.28; [−0.378–−0.167]; *p* < 0.01). In a similar fashion, BPQ-22 scores showed weak negative associations with anxiety subscale scores of the DASS-21 (DASS-21-A) (ρ = −0.12; [−0.222–−0.005]; *p* < 0.05). Thus, although the associations involving body perception were statistically significant, their magnitude was small. Finally, as expected, depression, anxiety, and stress subscale scores of the DASS-21 (i.e., DASS-21-D, DASS-21-A, DASS-21-S, respectively), and DASS-21 total scores (DASS-21-TOT) showed strong to very strong positive associations among themselves (ρ = from 0.60 to 0.95; *p* < 0.01). The complete 95% BCa bootstrap confidence intervals for the correlation coefficients are reported in Table S1.

### 3.3. Hierarchical Regression Analyses

Variance Inflation Factor (VIF) values were calculated for all predictors and ranged from 1 to 3.29, indicating no important evidence of multicollinearity [64]. Additional data inspections further suggested that the assumptions of linearity and homoscedasticity were adequately satisfied [65]. The findings from hierarchical multiple regression models predicting NPSS, SCS-SF, and BPQ-22 scores are reported in the following subsections, while an overall summary of these analyses is provided in Table 2. Confidence intervals are reported in Table S4.

#### 3.3.1. NPSS

In the hierarchical regression model predicting NPSS scores, the variables entered in step 1—namely DASS-21-D, DASS-21-A, and DASS-21-S subscale scores—did not account for a significant proportion of the variance, explaining 0.2% of the outcome variability (ΔR^2^ = 0.002, *p* = 0.865). Within this initial step, no DASS-21 subscale scores emerged as significant independent predictors. In step 2, the inclusion of BPQ-22 scores produced a significant increase in explained variance (ΔR^2^ = 0.046, *p* < 0.001), indicating that body perception contributed an additional 4.6% of variance to NPSS beyond that explained by depressive symptoms, anxiety symptoms, and stress symptom severity. In this step, only BPQ-22 scores (β = −0.22, *p* < 0.001) emerged as a significant predictor. In step 3, the inclusion of SCS-SF scores yielded a further significant increase in explained variance (ΔR^2^ = 0.224, *p* < 0.001), suggesting that self-compassion explained an additional 22.4% of the variance after controlling for all previously considered variables. In accordance with this, in step 3, only SCS-SF scores remained a significant individual predictor of NPSS (β = 0.49, *p* < 0.001), whereas neither BPQ-22 scores nor DASS-21 subscale scores retained statistical significance.

#### 3.3.2. SCS-SF

Regarding the hierarchical regression model predicting SCS-SF scores, in step 1, DASS-21-D, DASS-21-A, and DASS-21-S subscale scores did not account for a significant proportion of the variance, explaining 0.5% of the outcome variability (ΔR^2^ = 0.005, *p* = 0.672). In the first step, no DASS-21 subscale scores emerged as a significant independent predictor. In step 2, BPQ-22 scores were found to be a significant predictor of SCS-SF (β = −0.11, *p* < 0.001) and accounted for a significant increase in explained variance (ΔR^2^ = 0.078, *p* < 0.001), indicating that body perception contributed an additional 7.8% of variance in NPSS beyond that explained by depressive symptoms, anxiety symptoms, and stress symptoms severity. In step 3, the further inclusion of NPSS scores yielded an additional significant increase in explained variance (ΔR^2^ = 0.216, *p* < 0.001), suggesting that NPSS was able to explain an additional 21.6% of the variance after controlling for all previously entered variables. In step 3, both BPQ-22 (β = −0.18, *p* < 0.001) and NPSS (β = 0.48, *p* < 0.001) scores remained significant independent predictors.

#### 3.3.3. BPQ-22

The last hierarchical regression model with BPQ-22 scores as the dependent variable, step 1 showed that DASS-21-D, DASS-21-A, and DASS-21-S subscale scores did not account for a significant proportion of the variance, explaining 0.9% of the total variance (ΔR^2^ = 0.009, *p* = 0.404). At this stage, no DASS-21 subscale scores emerged as a significant independent predictor. In step 2, the inclusion of NPSS scores resulted in a significant increase in explained variance (ΔR^2^ = 0.045, *p* < 0.001), indicating that psychological safety accounted for an additional 4.5% of the variance of BPQ-22 scores after controlling for depressive symptoms, anxiety symptoms, and stress symptoms severity. In this step, only NPSS (β = −0.21, *p* < 0.001) scores remained as a significant predictor. In step 3, the inclusion of SCS-SF scores revealed a further significant increase in explained variance (ΔR^2^ = 0.039, *p* < 0.001), suggesting that self-compassion explained an additional 3.9% of the variance beyond that accounted for by psychological safety and DASS-21 subscales. In the final step, only SCS-SF (β = −0.23, *p* < 0.001) scores remained as a significant independent predictor.

### 3.4. Mediation Analysis

Body perception was found to show a statistically significant total effect on psychological safety (path c; β = −0.45, [−0.674–−0.222], *p* < 0.001, SE = 0.12). Considering the existence of a significant total effect between BPQ-22 and NPSS, and the fact that self-compassion remained the only significant predictor in a hierarchical regression model beyond NPSS and DASS-21 subscales, the role of SCS-SF was investigated as a possible mediator of the effects between BPQ-22 and NPSS. BPQ-22 scores were able to predict SCS-SF scores (path a; β = −0.34, [−0.474–−0.218], *p* < 0.001, SE = 0.07), in turn, SCS-SF were able to predict NPSS scores (path b; β = 0.87, [0.684–1.017], *p* < 0.001, SE = 0.09). After accounting for self-compassion and considering SCS-SF scores as a mediator, the direct effect of body perception on psychological safety, as measured by NPSS, showed no statistical significance (path c′; β = −0.16, [−0.360–0.053], *p* = 0.13, SE = 0.11). Overall, these results suggest that self-compassion may act as a total mediator of the effects between body perception and psychological safety, as measured by the NPSS.

Multi-group path analyses examined whether the mediation model was structurally equivalent across gender (Table S2) and educational groups (Table S3). Following recommendations by Ryu [60] and Ryu and Cheong [61], unconstrained models were compared with models in which all structural paths were constrained to equality, using likelihood-ratio tests with α = 0.05. The equality constraints did not significantly worsen model fit across gender, χ^2^(3) = 0.271, *p* = 0.965, CFI = 1.000, TLI = 1, RMSEA = 0.000, SRMR = 0.010, or educational groups, χ^2^(6) = 7.69, *p* = 0.262, CFI = 0.987, TLI = 0.981, RMSEA = 0.05, SRMR = 0.038. These findings supported the structural equivalence of the mediation paths across both grouping variables.

## 4. Discussion

**Figure 1:**
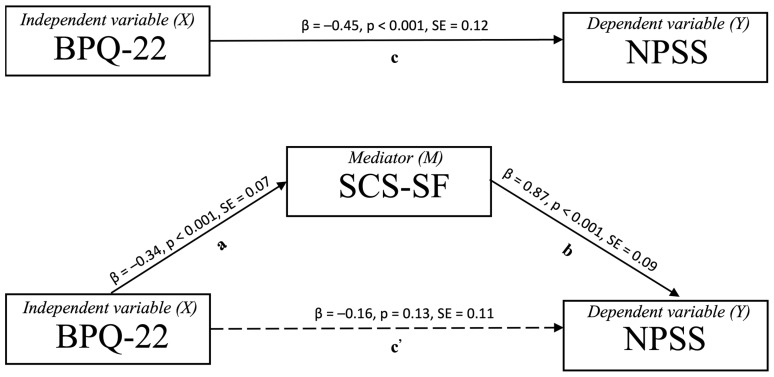
Mediation model indicating SCS-SF scores as a total mediator of the effects between BPQ-22 scores and NPSS scores.

The aim of the current study was to elucidate how self-compassion (SCS-SF scores) and body perception (BPQ-22 scores) relate to psychological safety, measured through the NPSS. Although body perception was associated with psychological safety during bivariate analyses, this association was not significant when self-compassion was considered in the model [66]. In accordance with this, self-soothing touch, as a conscious physical gesture associated with self-compassion, was able to reduce momentary stress, fatigue, and loneliness comparable to brief meditation [67]. This evidence is in line with previous evidence suggesting that strategies related to self-soothing touch are effective ways to promote individuals’ resilience against stress [68,69,70]. Furthermore, mediation analysis revealed that self-compassion scores were able to fully mediate the effects of body perception scores on psychological safety, as measured by the NPSS. Figure 1 and the hierarchical models shown in Table 2 suggest that self-compassion may be the process through which embodied experience of self-soothing becomes salient in terms of psychological safety [25,71,72].

Regarding Spearman correlations, NPSS scores were moderately and positively correlated to self-compassion, whereas their association with body perception was weaker and negative [25,73]. Self-compassion, in turn, was also weakly and negatively associated with body perception [22]. Conversely, depression, anxiety, and stress scores were strongly correlated, as expected, but showed no significant correlations with psychological safety or self-compassion in our sample. Though it has been reported that self-compassion may exert a negative direct effect on depressive symptoms measure with DASS-21 in the Iranian population [74], cultural differences may exist [75] and, considering that we administered the BPQ-22 whose wording of the items is biased towards a representation of unpleasant bodily feelings, it may have intercepted and explained the effects of depression, anxiety and stress [22,76]. Overall, this evidence suggests that psychological safety, as measured by the NPSS, may represent a specific domain involving regulation, perceived safety, and the intention to continue cultivating the ability to remain open to experience without becoming defensive. Considering that SCS-SF scores showed the strongest association with NPSS scores, this interpretation is consistent with our evidence [39].

Regression analyses highlighted that, when NPSS was considered as the dependent variable, symptoms of depression, anxiety, and stress were not able to explain any of its variance. BPQ-22 scores accounted for a small but significant amount of additional variance, indicating that body perception showed some predictive value. However, once SCS-SF scores were considered in the model, self-compassion was able to explain a much higher increase in variance, so that BPQ-22 was not able to show significant scores yet. Remarkably, the contribution of body perception to psychological safety may be influenced by how bodily states are worded, so that the SCS-SF emerged as a unique predictor over and beyond BPQ-22 and DASS-21 subscales [39,40]. Feeling unsafe in one’s own body may not depend on the bodily sensations themselves; rather, it may depend on the fact that those sensations are accompanied by a more critical, less kind, or less balanced way of relating to oneself [77]. As a matter of fact, several lines of research have shown that traumatic events, especially at an early age and in an interpersonal context, may deteriorate the experience of the body as a safe entity and promote self-criticism [30,39,40], while self-compassion levels suggest promoting a bodily self and a sense of safety for one’s own body [77,78].

Subsequently, considering self-compassion as the criterion variable and the main outcome, BPQ-22 significantly predicted SCS-SF scores, even after controlling for DASS-21 subscales. NPSS then added a substantial amount of variance, and in the final model, both body perception and psychological safety remained significant predictors of self-compassion. This is a useful result because it suggests that the three constructs may overlap, but not be interchangeable, and may retain a specific conceptual relationship. Self-compassion is clearly linked to psychological safety, but it is also tied to embodied experience [22,25,76]. While the NPSS is considered the dependent variable, SCS-SF is able to integrate the effects of the BPQ-22, resulting in the unique NPSS predictor. When the SCS-SF is considered the criterion variable, both the BPQ-22 and the NPSS remained as significant predictors. This effect may be explained by the fact that SCS-SF is influenced by both the actual body perception and by the concurrent sense of safety felt within one’s own body [39]. Finally, when BPQ-22 is considered as the criterion variable, NPSS initially predicted body perception, but this effect was no longer significant when self-compassion was entered into the model. Overall, these results suggest that when self-compassion is entered in the model, its effects are able to integrate the role of psychological safety and promote embodied safety.

Regarding mediation analysis, the total effect of body perception on psychological safety was significant, and BPQ-22 scores also significantly predicted self-compassion. Self-compassion, in turn, strongly predicted NPSS scores. Yet once self-compassion was included as a mediator, the direct effect of body perception on psychological safety was no longer significant. Remarkably, this result suggests that the pathway from the perception of safety in one’s own body to psychological safety may not be primarily direct but may be consistent with an important mediator, such as self-compassion, and not by other related, and partially overlapped constructs, such as self-esteem [79]. In fact, bodily sensations, autonomic reactivity, and the subjective perception of internal states may influence psychological safety mainly through a person’s capacity to respond to those experiences with kindness, emotional balance, and non-defensive awareness [80,81]. The multi-group analyses indicated that the structural relationships among body perception, self-compassion, and psychological safety did not differ significantly across gender or educational groups. This finding supports the cross-group stability of the proposed mediation process and suggests that self-compassion may represent a mechanism linking bodily experience to psychological safety.

The clinical implications are worth noting. These data suggest that interventions aimed at strengthening self-compassion could be particularly useful in people who report heightened bodily sensitivity or autonomic discomfort [82]. In populations characterized by hyperarousal, shame, persistent self-criticism, trauma-related defensiveness, or somatic vigilance, work on bodily awareness alone may not be enough [83,84]. Increasing awareness without changing the quality of self-relating might be insufficient for improving long-term psychological safety. Whether this interpretation also applies to PTSD symptom severity should be directly examined in trauma-exposed and clinically characterized samples [85]. Hence, self-compassion could represent a clinically modifiable target through which psychological safety becomes more accessible, even when bodily dysregulation may remain present. However, although body perception, self-compassion, and psychological safety are theoretically relevant to trauma-related functioning, trauma exposure and post-traumatic symptoms were not assessed in the present study. Therefore, the findings cannot be directly generalized to PTSD or complex trauma, and their clinical relevance for these conditions should be examined in specifically characterized samples.

The present results should be interpreted considering the following limitations. First, our study was cross-sectional, hence no causal direction can be established. Second, the study relied entirely on self-report measures, which may have inflated associations due to common method variance. In addition, social desirability, response-style effects, and common method variance may have contributed to the observed associations and may have partially inflated their magnitude. Although self-report measures are necessary for assessing subjective experiences such as psychological safety, self-compassion, and body perception, future research should adopt multimethod designs combining questionnaires with objective psychophysiological indices, including heart rate variability, heart rate, respiratory measures, and electrodermal activity. Where theoretically appropriate, endocrine or other biological indicators could also be included [86,87]. Such an approach would clarify the convergence between perceived psychological safety and autonomic regulation and provide a more comprehensive assessment of the proposed model. Third, our study sample was a convenience sample and may not fully represent more symptomatic clinical populations. Fourth, although the NPSS and the SCS-SF assess theoretically distinct constructs, some conceptual proximity should be acknowledged. In particular, the compassion dimension of the NPSS may share variance with self-compassion-related processes, potentially contributing to the magnitude of their association. However, the NPSS primarily captures safety across interpersonal, compassionate, and bodily domains, whereas the SCS-SF assesses self-directed responding to personal distress. Previous psychometric evidence showed only a moderate association between the two measures, supporting their related but non-redundant nature [22]. Fifth, the associations between body perception, psychological safety, and self-compassion were statistically significant but small in magnitude. Accordingly, these findings should be interpreted with caution. The clinical relevance of these associations should be confirmed in longitudinal, experimental, and clinically characterized samples. Sixth, although depression, anxiety, and stress were included as covariates, other residual confounder variables cannot be excluded. Traumatic history, previous or ongoing psychotherapy, medical and psychiatric conditions, psychotropic medication use, mindfulness, and personality traits were not systematically assessed and may partly account for the observed associations among body perception, self-compassion, and psychological safety. Finally, the sample characteristics may reduce generalizability, and replication in more heterogeneous or clinically severe groups would be important.

Despite these limitations, our study may help to clarify the understanding of psychological and bodily determinants underlying psychological safety. Our findings support the idea that psychological safety may not be simply explained by lower bodily dysregulation or by lower emotional distress, but that the effect of body perception on psychological safety may depend on whether individuals are able to approach their own internal experience with compassion. Future longitudinal studies should test whether changes in self-compassion precede changes in psychological safety, and whether interventions focused on compassionate self-relating can reduce the negative impact of dysregulated body perception on perceived safety. If confirmed, this line of work may help refine psychophysiological models of safety by showing that the body matters, but that how one treats oneself in relation to the body may matter even more.

## Figures and Tables

**Table 1:** Descriptive statistics and Spearman correlations among the study measures (n = 332).

Measure	M	SD	Med	IQR	Range	α	1	2	3	4	5	6
1. NPSS	112.17	15.40	114	19	64–145	0.92						
2. SCS-SF	40.32	8.83	41	12	14–60	0.89	0.49 **					
3. BPQ-22	33.19	7.13	32	11.75	22–60	0.86	−0.20 **	−0.28 **				
4. DASS-21-D	6.04	5.10	4	7	0–21	0.92	−0.07	−0.05	−0.08			
5. DASS-21-A	3.47	4.14	2	5	0–20	0.86	0.04	0.01	−0.12 *	0.60 **		
6. DASS-21-S	9.06	5.39	8.5	8	0–21	0.87	−0.06	−0.07	−0.10	0.78 **	0.74 **	
7. DASS-21-TOT	18.58	13.12	15.50	18	0–58	0.95	−0.05	−0.05	−0.11	0.90 **	0.82 **	0.95 **

Note: M = Mean score; SD = Standard deviation; Med = Median; IQR = Interquartile range; α = Cronbach’s alpha; NPSS = Neuroception of Psychological Safety Scale; SCS-SF = Self-Compassion Scale-Short Form; BPQ-22 = Body Perception Questionnaire-22; DASS-21-D = Depression Anxiety and Stress Scale-21-Depression subscale; DASS-21-A = Depression Anxiety and Stress Scale-21-Anxiety subscale; DASS-21-S = Depression Anxiety and Stress Scale-21-Stress subscale; DASS-21-TOT = Depression Anxiety and Stress Scale-21-Total score. * *p* < 0.05, ** *p* < 0.01.

**Table 2:** Hierarchical multiple regression analyses predicting NPSS, SCS-SF, and BPQ-22 scores (n = 332).

Predictor	Model 1	Model 2	Model 3
Criterion: NPSS			
Δ*R*^2^	0.002	0.046 ***	0.224 ***
DASS-21-D	−0.01 (0.26)	−0.01 (0.26)	0.01 (0.23)
DASS-21-A	0.04 (0.30)	0.04 (0.29)	0.02 (0.26)
DASS-21-S	−0.06 (0.29)	−0.09 (0.28)	−0.03 (0.25)
BPQ-22		−0.22 (0.12) ***	−0.08 (0.11)
SCS-SF			0.49 (0.09) ***
Criterion: SCS-SF			
Δ*R*^2^	0.005	0.078 ***	0.216 ***
DASS-21-D	−0.03 (0.15)	−0.02 (0.14)	−0.02 (0.13)
DASS-21-A	0.05 (0.17)	0.06 (0.17)	0.04 (0.14)
DASS-21-S	−0.08 (0.16)	−0.11 (0.16)	−0.07 (0.14)
BPQ-22		−0.28 (0.07) ***	−0.18 (0.06) ***
NPSS			0.48 (0.03) ***
Criterion: BPQ-22			
Δ*R*^2^	0.009	0.045 ***	0.039 ***
DASS-21-D	0.01 (0.12)	0.01 (0.12)	0.01 (0.12)
DASS-21-A	0.01 (0.14)	0.02 (0.14)	0.03 (0.13)
DASS-21-S	−0.11 (0.13)	−0.12 (0.13)	−0.13 (0.13)
NPSS		−0.21 (0.03) ***	−0.10 (0.03)
SCS-SF			−0.23 (0.05) ***

Note 2. Standard errors in parentheses; NPSS = Neuroception of Psychological Safety Scale; SCS-SF = Self-Compassion Scale-Short Form; BPQ-22 = Body Perception Questionnaire-22; DASS-21-D = Depression Anxiety and Stress Scale-21-Depression subscale; DASS-21-A = Depression Anxiety and Stress Scale-21-Anxiety subscale; DASS-21-S = Depression Anxiety and Stress Scale-21-Stress subscale. *** *p* < 0.001.

## Data Availability

The data presented in this study are available on request from the corresponding author. The data are not publicly available due to privacy issues.

## Supplementary Materials

The following supporting information can be downloaded at: https://www.mdpi.com/article/10.3390/jcm15135177/s1. Table S1: Confidence intervals (95%) of Spearman correlations among the study measures (n = 332); Table S2: Multi-Group mediation model showing structural invariance across gender on the total sample (n = 332); Table S3: Multi-Group mediation model showing structural invariance across education on the total sample (n = 332); Table S4: Confidence intervals of hierarchical multiple regression analyses predicting NPSS, SCS-SF, and BPQ-22 scores (n = 332).
