# Supplementary material for: The Role of Self-Compassion and Body Perception in Predicting Psychological Safety
Source: J Clin Med. 2026 Jul 2;15(13):5177. doi: 10.3390/jcm15135177 (PMC13363664; doi:10.3390/jcm15135177)
Supplement: Supplementary file 1 [file jcm-15-05177-s001.zip › jcm-4358222-supplementary.pdf]

Supplementary Materials

Table S1

Confidence intervals (95%) of Spearman correlations among the study measures (n = 332).

Measure	1	2	3	4	5	6
1. NPSS						
2. SCS-SF	.397 – .573**					
3. BPQ-22	-.301 – -.085 **	-.378 – -.167**				
4. DASS-21-D	-.177 – .034	-.156 – .054	-.195 – .027			
5. DASS-21-A	-.072 – .137	-.106 – .108	-.222 – -.005*	.525 – .668**		
6. DASS-21-S	-.165 – .050	-.169 – .033	-.201 – .020	.725 – .822**	.684 – .791**	
7. DASS-21-TOT	-.152 – .057	-.157 – .053	-.223 – .004	.867 – .920**	.780 – .857**	.936 – .961**

Note: NPSS = Neuroception of Psychological Safety Scale; SCS-SF = Self-Compassion Scale-Short Form; BPQ-22 = Body Perception Questionnaire-22; DASS-21-D = Depression Anxiety and Stress Scale-21-Depression subscale; DASS-21-A = Depression Anxiety and Stress Scale-21-Anxiety subscale; DASS-21-S = Depression Anxiety and Stress Scale-21-Stress subscale; DASS-21-TOT = Depression Anxiety and Stress Scale-21-Total score.

*$p < .05$, ** $p < .01$

Table S2.

Multi-Group mediation model showing structural invariance across gender on the total sample (n = 332).

Model	χ^2	*p*	df	CFI	TLI	RMSEA	SRMR	PCLOSE
Unconstrained model	0.000	—	0	1	1	0	0	—
Constrained model	0.271	0.965	3	1	1	0	0.01	0.981

Note: χ^2 = chi-square value; *p* = *p*-value; df = degrees of freedom; CFI = Comparative Fit Index; TLI = Tucker Lewis Index; RMSEA = Root Mean Square Error of Approximation; SRMR = standardized root mean residual; PCLOSE = *p*-value of close fit.

Table S3.

Multi-Group mediation model showing structural invariance across education on the total sample (n = 332).

Model	χ^2	*p*	df	CFI	TLI	RMSEA	SRMR	PCLOSE
Unconstrained model	0.000	—	0	1	1	0	0	—
Constrained model	7.69	0.262	6	0.987	0.981	0.05	0.038	0.424

Note: χ^2 = chi-square value; *p* = *p*-value; df = degrees of freedom; CFI = Comparative Fit Index; TLI = Tucker Lewis Index; RMSEA = Root Mean Square Error of Approximation; SRMR = standardized root mean residual; PCLOSE = *p*-value of close fit.

Table S4

Confidence intervals of hierarchical multiple regression analyses predicting NPSS, SCS-SF and BPQ-22 scores (n = 332).

Predictor	Model 1	Model 2	Model 3
Criterion: NPSS			
DASS-21-D	-.737 – .385	-.519 – .490	-.424 – .460
DASS-21-A	-.436 – .740	-.416 – .734	-.447 – .562
DASS-21-S	-.539 – .492	-.793 – .306	-.576 – .388
BPQ-22		-.694 – -.233***	-.375 – .045
SCS-SF			.693 – 1.031***
Criterion: SCS-SF			
DASS-21-D	-.340 – .250	-.322 – .246	-.283 – .215
DASS-21-A	-.224 – .449	-.206 – .442	-.209 – .358
DASS-21-S	-.444 – .198	-.483 – .136	-.378 – .164
BPQ-22		-.476 – -.217***	-.336 – -.104***
NPSS			.220 – .327***
Criterion: BPQ-22			
DASS-21-D	-.219 – .257	-.216 – .250	-.219 – .238
DASS-21-A	-.256 – .287	-.235 – .296	-.218 – .304
DASS-21-S	-.405 – .113	-.417 – .090	-.425 – .072
NPSS		-.148 – -.050***	-.100 – .012
SCS-SF			-.283 – -.087***

Note: NPSS = Neuroception of Psychological Safety Scale; SCS-SF = Self-Compassion Scale-Short Form; BPQ-22 = Body Perception Questionnaire-22; DASS-21-D = Depression Anxiety and Stress Scale-21-Depression subscale; DASS-21-A = Depression Anxiety and Stress Scale-21-Anxiety subscale; DASS-21-S = Depression Anxiety and Stress Scale-21-Stress subscale.

* = $p < .05$; ** $p < .01$; *** $p < .001$
